# Patient’s perception of kidney stone prevention within the emergency department and its adherence factors: a single institution study

**DOI:** 10.1186/s12873-019-0263-0

**Published:** 2019-09-02

**Authors:** Mohamad Moussa, Mohamed Abou Chakra

**Affiliations:** 1 Head of Urology Department, Zahra Hospital, University Medical Center, Lebanese University, Beirut, Lebanon; 2 Department of Urology, Lebanese University, Beirut, Lebanon

**Keywords:** Emergency department, Kidney stones, Prevention, Adherence

## Abstract

**Background:**

No known data in the literature assessing practice of kidney stone prevention in the emergency department (ED) is available.

**Objectives:**

Assess patient perception and compliance to kidney stone prevention given within the emergency department. It also indirectly detects the attitude and practice patterns of primary care providers in kidney stone prevention.

**Materials and methods:**

This is a qualitative study done in a single institution from January 2018 to January 2019 that includes 99 patients that were diagnosed with kidney or ureteral stone in ED and were discharged home, all of them where stone formers. They were asked to fill a self- administered questionnaire when they are able to read, or interviewed by the resident within the ED when they are unable to read.

**Results:**

The majority of patients (68%) did not receive any instructions about kidney stones prevention within the ED. Most of patients who follow instructions if it was given were educated (90%), had an insurance coverage (85%), and had an income higher than $1000 per month (76%), (*p* < 0.05).

Seventy one percents of patients believe in the effectiveness of stone prevention if it was provided and most of them are interested in learning about these preventive strategies (82%).

Reasons for not following the instructions about kidney stones prevention measures were the cost (53.1%) following by the lack of explanation by ED physicians (18.8%).

The majority of patients (62.6%) prefer to receive kidney stones prevention measures from urologists.

**Conclusion:**

Most of patients in our institute did not receive kidney stones prevention measures in ED despite that they declared their interest in following these measures. Most of the time they did not adhere to those measures due to socioeconomic factors and lack of clarifications. If these instructions were given within the ED, it could lead to an acceptable compliance rate.

**Electronic supplementary material:**

The online version of this article (10.1186/s12873-019-0263-0) contains supplementary material, which is available to authorized users.

## Background

Kidney stones are becoming a ubiquitous problem in the emergency setting as both prevalence and incidence are increasing in all age groups [1]. Urinary tract stones can be classified according to their composition, location, size, etiology, radiological characteristics, and risk of recurrence. About 80% of patients predominantly have calcium oxalate and/or calcium phosphate stones. Uric acid and struvite stones each account for 5–10% and cystine stones are quite rare [2]. Emergency department (ED) visits for patients with kidney stones are common, as most of them are discharged from the ED but little of them are followed after discharge. A large study conducted in the US shows 11% of patients with kidney stone had additional visits to the ED; revisit depends on medical and non-medical factors such as patient coverage and access to urologic care [3]. There is dietary and medical therapy for kidney stones prevention such as increasing daily fluid intake, following a balanced diet that is rich in vegetables and fibers, normal calcium content with a limitation of sodium chloride content, and limited animal protein content. Fluid intake should be increased to achieve a daily urine output of 2.0–2. 5 liters. A low-normal protein intake decrease calciuria and could be useful in stone prevention and preservation of bone mass. Omega-3 fatty acids decrease calciuria, but their impact on the urinary stone risk profile is uncertain. The DASH-style(Dietary Approaches to Stop Hypertension) diet that is rich in fruits and vegetables, moderate in low-fat dairy products and low in animal proteins and salt is associated with a marked decrease of incidence of stone formation [4–7]. A recent meta-analysis, based on the results of 9 studies (2 Randomised clinical trials and 7 observational studies), concluded that high fluid consumption significantly reduces the risk of incident and recurrent kidney stones. Although increased water intake appears to be safe, more studies assessing the safety of high fluid intake to prevent kidney stones are needed especially in patients with a high risk of volume overload or hyponatremia [8].

It is very important after initial evaluation of patients in the ED to follow these patients after discharge, secondary prevention of kidney stones not only decrease the patient revisit to ED but can reduce the healthcare cost and complications of kidney stones [9]. The most common type of kidney stones in Lebanon is calcium oxalate stones while the least common are cystine stones. The main preventive measures for kidney stone in Lebanon are mostly dietary modifications; no clear data for the secondary prevention of kidney stones in Lebanon [10]. There is no known data in the literature assessing the practice concerning kidney stone prevention before patient discharge from the ED.

The primary objective of this study is to assess how the patients perceive kidney stones prevention measures if they are given in the ED. The secondary objective is to evaluate how the patients are following the instructions that were given before discharge and to suggest solutions for a better compliance rate. Our study was also implemented as a qualitative study to indirectly detect the attitude and practice patterns of primary care providers in kidney stone prevention.

## Materials and methods

This is a qualitative study on the current prevention instructions and education concerning kidney stones given to patients in the ED at Zahra Hospital at Beirut. The study was approved by our Institutional Review Board. We interviewed patients in the ED that were diagnosed with recurrent kidney or ureteral stones from January 2018 to January 2019. During this time frame, 99 patients were enrolled in the study, all of them agreed to participate. Symptomatic patients with Kidney or ureteral stones were diagnosed by non-contrast-enhanced computed tomography (CT).

All patients were asked to fill a self-administered questionnaire (Additional file 1). The questionnaires were available in 3 languages: Arabic, English and, French and taking less than 3 min to be filled. If the patient is unable to read, the resident within the ED read the questions for him and recorded the answers after taking his consent. These responses were analysed by both authors later on. The participants provide the information with no feedback on the findings later on.

Detailed information were elicited from the patients using these questionnaires regarding gender, educational level, socioeconomic status, number of emergency visits for renal colics, knowledge about kidney stone prevention, instructions given to patients by ED physicians about stones prevention and patient’s perception about these instructions, factors that affect their adherence to these recommendations. The exclusion criterion was first time stone former. The survey was completed before patient discharge to home, it was distributed to patients by the nurse before discharge if they can read or presented by the resident within the ED, without the involvement of ED physicians during the study period.

After reviewing the literature related to kidney stones prevention, a questionnaire format was designed by many urologists based on the current practice guidelines with subspecialty training in stone management before recruitment. Due to the lack of studies addressing kidney stone prevention within the ED, no validated questionnaires were used.

The study was enrolled at Zahra Hospital in Beirut which is an academic, tertiary care hospital with 300 beds. The ED contains 18 beds; it is staffed by 15 emergency medicine physicians. The urology department contains 20 beds; it is staffed by 8 urologists.

Data analysis was mainly descriptive, targeting the patient’s perception of kidney stones prevention measures as well as the differences of these perceptions between those who receive and those who do not receive instructions, and between those who are willing and those who are not willing to follow the instructions. Data analysis was performed using R software. In all analyses, a *P value* < 0.05 was considered significant. The tests used included the Pearson’s Chi-squared test for dichotomous or multinomial qualitative variables; and when the expected values within cells were < 5, the Fisher’s Exact test was used.

A multiple stepwise forward logistic regression was performed to evaluate the patient variables associated with the fact of following instructions about kidney stones prevention. All variables having *P*-value ≤0.2 in simple bivariate logistic regression were considered in the multiple logistic regressions. Models that accounted for all these variables and that showed significant associations were retained.

Models adequacy to data was insured by the Hosmer–Lemeshow test. Non significant (*P*- value > 0.05) was a condition to test the goodness of fit of the model and its ability to predict the dependent variable (willingness to follow instructions). The Nagelkerke R2 was checked to assess the usefulness of the explanatory variables in predicting the dependent variable. The contribution of each determinant in the multivariable analysis was expressed as an odds ratio (OR) and a 95% confidence interval (CI).

## Results

### Participant characteristics

The majority of patients (68%) did not receive any instructions about kidney stones prevention before emergency room discharge. Table 1 summarizes the characteristics of patients in the two groups of the study population: patients who receive instructions about kidney stones prevention when they were discharged from the ED, and patients who did not receive any instructions.

**Table 1:** Characteristics of patients, n (%)

Characteristic	Overall (*N* = 99)	Receive instructions: No (*n* = 67)	Receive instructions: Yes (*n* = 32)	*P value*
Gender				
Male	57 (57.6)	38 (56.7)	19 (59.4)	0.974*
Female	42 (42.4)	29 (43.3)	13 (40.6)	
Age				
[10-20[	12 (12.1)	8 (11.9)	4 (12.5)	0.463**
[20-30[	24 (24.2)	17 (25.4)	7 (21.9)	
[30–40[	25 (25.3)	20 (29.9)	5 (15.6)	
[40–50[	22 (22.2)	13 (19.4)	9 (28.1)	
[50–60[	16 (16.2)	9 (13.4)	7 (21.9)	
Working Situation				
Non-working	22 (22.2)	14 (20.9)	8 (25)	0.841*
Working	77 (77.8)	53 (79.1)	24 (75)	
Educational Level				
Illiterate	20 (20.2)	19 (23.4)	1 (3.1)	0.026**
Primary education	16(16.2)	10 (14.9)	6 (18.8)	
Intermediate education	29 (29.3)	17 (25.4)	12 (37.5)	
High school	19 (19.2)	13 (19.4)	6 (18.8)	
University	15 (15.2)	8 (11.9)	7 (21.9)	
Medical Coverage				
No	25 (25.3)	21 (31.3)	4 (12.5)	0.076**
Yes	74 (74.7)	46 (68.7)	28 (87.5)	
Monthly Salary				
<$500	16(6.2)	14 (20.9)	2 (6.3)	0.316**
[$500–$1000[	15 (15.2)	10 (14.9)	5 (15.6)	
[$1000–$2000[	44 (44.4)	28 (41.8)	16 (50)	
>$2000	24 (24.2)	15 (22.4)	9 (28.1)	
Economical Status				
Low - Salary <$1000	31 (31.3)	24 (35.8)	7 (21.9)	0.243*
High - Salary >$1000	68 (68.7)	43 (64.2)	25 (78.1)	
Number of ER visits				
2 times	34 (34.3)	25 (37.3)	9 (28.1)	0.433**
3 to 5 times	59 (59.6)	37 (55.2)	22(68.8)	
> 5 times	6 (6.1)	5 (7.5)	1 (3.1)	

*Pearson’s Chi-squared test; **Fisher’s Exact Test

Level of education was significantly different between patients who receive and who do not receive instructions from physicians (*p* < 0.05).

Gender, age, working situation, income, insurance status, and the number of ED visits of patients were not significantly associated with receiving instructions from ED physicians.

Most of patients who follow instructions (62%) if it was given are educated (90% vs. 10%), had an insurance coverage (85% vs. 15%), and had an income higher than $1000 per month (76 vs. 14%), than those who do not follow instructions (*p* < 0.05).

Gender, age, working situation, and the number of ED visits were not significantly associated with following instructions or not.

### Patient’s perception and knowledge of kidney stone prevention

Twenty-six percents of patients who were asked about their knowledge of kidney stones prevention measures had no idea about this topic, but 71% of patients believe in the effectiveness of those measures if they are given and most of them showed interest in learning about prevention strategies (82%). The knowledge of patients, the perception of the effectiveness of prevention measures, and the interest of learning more about kidney stones prevention were significantly different between patients who receive and who do not receive instructions from physicians (*p* < 0.05).

If patients had no interest and no idea about kidney stones prevention, there is a high risk of not receiving instructions (respectively 38.8% vs. 0 and 25.4% vs. 0%); however, most patients who believe in the effectiveness of prevention measures receive instructions (respectively 96.9% vs. 59.7 and 98.6% vs.3.6%). Results are summarized in Table 2.

**Table 2:** Patients’ perception of kidney stones prevention measures, n (%)

Perception	Overall (*N* = 99)	Receive instructions: No (*n* = 67)	Receive instructions: Yes (*n* = 32)	*P value*
Prevention knowledge				
Dietary recommendations	41 (41.4)	20 (29.9)	21(65.6)	<0.001**
Effective measure to prevent stone recurrence	17(17.2)	12 (17.9)	5 (15.6)	
Medications given	15 (15.2)	9 (13.4)	6 (18.8)	
No idea	26 (26.2)	26 (38.8)	0 (0)	
Belief in the effectiveness of prevention measures				
No	28 (28.3)	27 (40.3)	1 (3.1)	0.0003*
Yes	71 (71.7)	40 (59.7)	31 (96.9)	
Interest to learn about prevention measures				
No	17 (17.2)	17 (25.4)	0 (0)	0.004*
Yes	82 (82.9)	50 (74.6)	32 (100)	

**Fig. 1:**
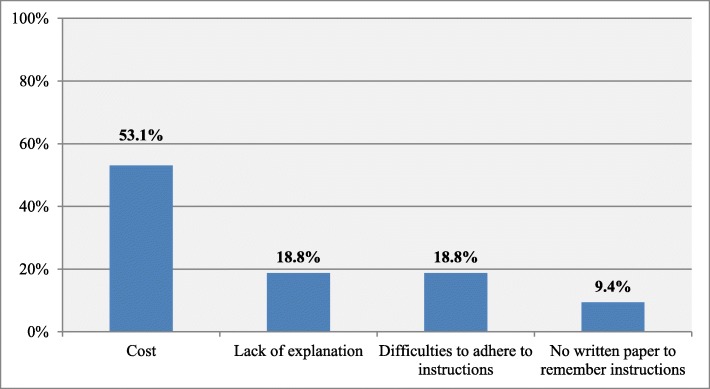
Reasons to not follow instructions among patients who receive instructions (*n* = 32)

The most common reason for not obeying instructions about kidney stones prevention measures was the cost (53. 1%) following by the lack of explanation by ED physicians and difficulties to adhere to instructions (18.8%). Results are illustrated in Fig. 1.

### Interventions to improve prevention measures according to patients suggestions

**Fig. 2:**
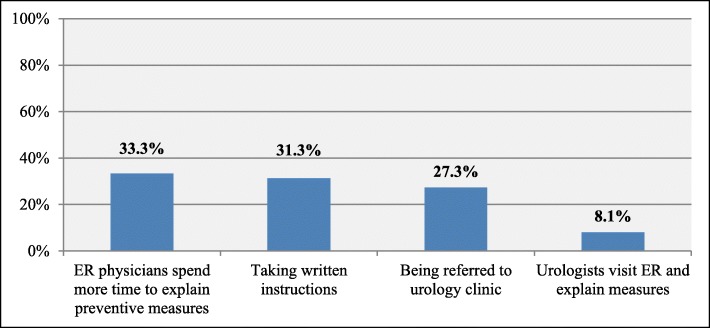
Patient’s suggestions to improve kidney stones prevention measures (*n* = 99)

Most of the patients believe that kidney stones prevention measures in ED will be improved if ED physicians spend more time explaining preventive measures (33.3%) and providing them with written instructions (31.3%), and by being referred to urology clinic after discharge(27.3%). Results illustrated in Fig. 2.

**Fig. 3:**
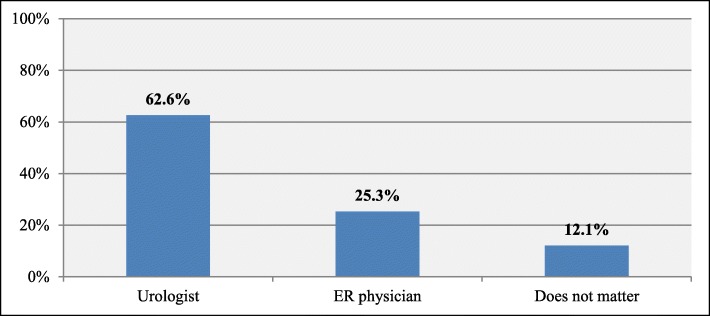
Physicians’ specialty preferred by the patients to give kidney stone prevention measures (*n* = 99)

Moreover, the majority of them prefer to receive kidney stone prevention measures from Urologists (62.6%) see Fig. 3.

## Discussion

The lifetime prevalence of nephrolithiasis in the United States is estimated to be between 5% and 12%, with the probability of having a stone varying according to age, gender, race, and geographic location [11]. Nephrolithiasis typically affects adult men more commonly than adult women, with a male to female ratio of 2 or 3: 1 [12]. About half of all stone-formers have one recurrence during their lifetime. The recurrence rate is high in 10–15% of all stone-formers, depending on the stone type and the severity of the disease [13]. For frequent stone formers, and even for some first-time stone formers, a metabolic evaluation is necessary. The urologist, rather than members of other medical specialties, seems to have the primary responsibility of stone management and prevention. All stone formers should follow preventive measures focusing on dietary habits by reducing animal protein intake, limiting dietary oxalate only if the patient has hyperoxaluria, consuming fruit juice, targeting calcium intake to 1,200 mg/day, reduced soft drinks intake [14–17].

More than 90% of patients evaluated in EDs for stones are discharged after treatment [18]. Preventing ED repeated visits is important because those visits contribute to increasing healthcare expenditures [19].

A study identified the incidence of follow-up in patients who attended an ED for kidney stones. It shows only 48% of patients seen in the ED for kidney stones received follow-up care, in 68.3% of these cases with a urologist. Among patients who received follow-up care, the use of stone prevention strategies was higher when the care was delivered by a urologist [20].

We found in our study that most of the patients (68%) did not receive any instructions about kidney stones prevention. Those who received instructions had an acceptable level of education. This may be related to two factors; first, the fact that educated patient will read more thoroughly about kidney stone disease and ask for clarifications about preventive measures, while on the other hand the ED physicians assumed most of the time that kidney stones prevention in illiterate patients is not important.

The instruction adherence to recommendations if it were given was 62% and most of the patients who followed these instructions were educated, had insurance coverage and an income more than 1000$ per month. This indicates that the patient socioeconomic status affects patient adherence to these instructions. A study of 300 patients evaluating compliance of the recurrent renal stone former with current Canadian Urological Association (CUA) best practice guidelines showed that 45.8% of patients were compliant with CUA best practice guidelines even if they received satisfactory education from their urologist and primary care physician. In addition, 67. 1% of patients believed in the efficacy of preventative stone measures and 22.8% of patients perceived their stone disease to be severe [21]. Dauw et al. found an adherence rate of 30.3% with preventive pharmacologic therapy in kidney stone patients. Female gender, less generous health insurance, had a higher probability of low adherence [22].

The lack of explanation and not providing written instructions can have an important role in adherence to the recommendations. Chan et al. concluded that people had poor knowledge of kidney stones, its suitable diet and the importance of more liquid consumption to prevent recurrence [23].

Most of the patients in our study were highly motivated to learn about kidney stones prevention and showed a high adherence rate if those instructions are given within the ED.

Many suggestions were made to improve kidney stones prevention strategies within the ED, highlighting the importance to give more time to patients for further explanation, urologist visit to ED for primary education as most of the patients prefer to receive kidney prevention recommendations from the urologist.

At the end of the study, we asked our ED physicians about the reasons for not giving instructions for patients before discharge. From fifteen ED physicians in our hospital that were asked, six of them said that the cause of not giving instructions was the lack of communication with the urologists and nephrologists concerning kidney stone prevention, five of them said that the cause was a shortage of time to explain and four of them said they lack the experience in such practice within the ED. We think that a multidisciplinary approach between Urologists, nephrologists and ED physicians in kidney stones prevention in the ED settings will help in the prevention of recurrent kidney stones disease. Collaborative care among physicians should be the aims of future study regarding kidney stones prevention.

Our study had several limitations. It describes a small number of patients at a single academic institution, thus potentially limiting its generalization to other populations. Since there were no previous studies to refer to, regarding kidney stone prevention in the primary care setting, we set a 1 year period to study this subject, where we studied 99 patients. The questionnaire distributed to participants was not validated; however, many urologists with subspecialty training in stone management formally reviewed the survey prior to recruitment.

## Conclusion

Most of the patients seen acutely in the ED for kidney stones do not receive prevention instructions by the ED physicians. If these instructions were given within the ED, it could lead to a high compliance rate. Many factors such as the socioeconomic status and medical coverage should be considered when kidney stones prevention tips were given.

## Additional file

Additional file 1: Patients questionnaires. (DOCX 17 kb)

## Data Availability

The datasets used and analyzed during the current study are available from the corresponding author on reasonable request.

## Abbreviation

ED: emergency department
