# Supplementary material for: Patient’s perception of kidney stone prevention within the emergency department and its adherence factors: a single institution study
Source: BMC Emerg Med. 2019 Sep 2;19:48. doi: 10.1186/s12873-019-0263-0 (PMC6719350; doi:10.1186/s12873-019-0263-0)
Supplement: Supplementary file 1 — Patients questionnaires. (DOCX 17 kb) [file 12873_2019_263_MOESM1_ESM.docx]

Additional file 1 -Patients questionnaires

1- Gender:

a- Male

b- Female

2- Date of Birth: Year of Birth

3- Are you working?:

a- No b- Yes

4- What is your educational level:

a- Illiterate b- Primary Education c- Intermediate Education

d-High School e- University

5- Do you have any medical coverage? :

a- No

b- Yes

6- How much is your salary? :

a- less than 500$

b- 500-1000$

c- 1000-2000$ d- more than 2000$

7- How many times did you visit the ER previously for kidney stone

a-Once b- Twice c- 3-5 d-more than 5 times

8-. What do you know about kidney stones prevention?

a-it is an effective measure to prevent stone recurrence

b-it is mainly dietary recommendations

c-it is mainly medications given

d-no idea

e-others

9-when you were discharged from the ER after the diagnosis of kidney stones and medical treatment was given ,did the ER physicians give you any instructions about kidney stones prevention ?

a-yes

b-no

10- if the ER physicians gave you instructions about stones prevention ,did/do you follow those instructions ?

a-yes

b-no

11-do you think that kidney stones prevention measures will affect your disease if it was given?

a-yes

b-no

12- Would you follow instructions if they were given?

a-yes

b-no

13-do you prefer that the urologists give to you kidney stone prevention instructions instead of the ER physician ?

a-yes

b-no

c-does not matter

14-if instructions are given to you and you don't follow it, what was the cause?

a-lack of explanation

b-cost

c-no written paper was given to remember those instructions

d-other

e-difficulties to adhere to it

15-are you interested to learn about kidney stone prevention?

a-yes

b-no

16-what do you suggest to improve secondary prevention of kidney stone in the ER?

1-ER physicians spend more time to explain those preventive measures

2- give me written instructions

3-urologist visits the ER and explain the measures

4- to refer me to the urology clinic.
